# Epidemiologic data and pathogen genome sequences: a powerful synergy for public health

**DOI:** 10.1186/s13059-014-0538-4

**Published:** 2014-11-18

**Authors:** Yonatan H Grad, Marc Lipsitch

**Affiliations:** Center for Communicable Disease Dynamics, Department of Epidemiology, Harvard School of Public Health, Boston, MA 02115 USA; Department of Immunology and Infectious Diseases, Harvard School of Public Health, Boston, MA 02115 USA; Division of Infectious Diseases, Brigham and Women’s Hospital, Harvard Medical School, Boston, MA 02115 USA

## Abstract

Epidemiologists aim to inform the design of public health interventions with evidence on the evolution, emergence and spread of infectious diseases. Sequencing of pathogen genomes, together with date, location, clinical manifestation and other relevant data about sample origins, can contribute to describing nearly every aspect of transmission dynamics, including local transmission and global spread. The analyses of these data have implications for all levels of clinical and public health practice, from institutional infection control to policies for surveillance, prevention and treatment. This review highlights the range of epidemiological questions that can be addressed from the combination of genome sequence and traditional ‘line lists’ (tables of epidemiological data where each line includes demographic and clinical features of infected individuals). We identify opportunities for these data to inform interventions that reduce disease incidence and prevalence. By considering current limitations of, and challenges to, interpreting these data, we aim to outline a research agenda to accelerate the genomics-driven transformation in public health microbiology.

## Introduction

**Figure 1:**
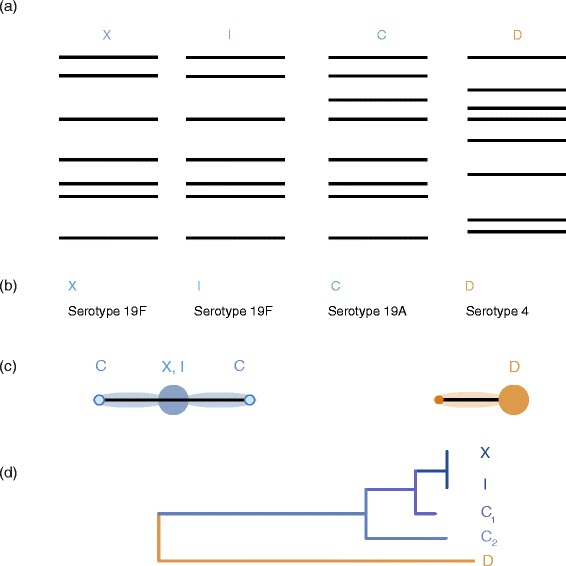
**Comparison of resolution of typing techniques.** Typing methods range in resolution, from low resolution, which can classify isolates as indistinguishable (I) from the index case (X), closely related (C, C_1_, and C_2_) or very different (D), to the high-resolution method of genome sequencing, which can distinguish isolates by single nucleotide variation. Isolates indistinguishable by lower-resolution techniques may be distinguishable by their sequences; indistinguishable by complete whole genome sequencing is by definition having the identical sequence. **(a-d)** Schematic representations of pulsed-field gel electrophoresis (PFGE) (a), seroptying (using the example of serotypes of *Streptococcus pneumoniae*) (b), multilocus sequence typing (MLST; in cartoon eBURST figure) (c), and a phylogeny from whole genome sequencing (d) show the different levels of resolution. Whereas in PFGE, serotype and MLST, isolates can be identified as at coarse levels of relatedness, genotyping offers higher-resolution typing. An isolate seen as closely related (C_1_) to the index case (X) in whole genome sequencing may be indistinguishable (I) in the first three methods, whereas a more distantly related isolate, as seen by whole genome sequencing (C_2_), might appear as closely related. Moreover, as described in the text, the integration of sequencing with molecular evolutionary theory provides much greater opportunity for phylogenetic inference, offering conceptual leaps beyond other typing methods and greater contributions to infectious disease epidemiology.

Infectious disease epidemiologists study patterns of disease incidence, and seek ways to turn observations about which individuals and populations become infected into strategies to decrease the burden of disease. The effort to identify predictors of who gets infected and who among these becomes symptomatic requires first and foremost the ability to define the disease. The advent of cheap, rapid whole-genome sequencing of pathogens is the latest in a historic progression of the ways in which epidemiologists classify disease; classification methods have progressed from clinical and epidemiological definitions of syndromes to microbiologic characterization of pathogens from infected individuals (Figure 1), and now to the use of pathogen genotype and genome sequence. Improved characterizations of pathogens and deeper understanding of their biology have driven the development of diagnostic techniques, vaccines and therapies, and have helped guide strategies for maximizing the impact of these tools for disease control and treatment. An example of this progression can be seen in the study of influenza, from the identification of influenza virus as the etiologic agent [1,2], whereas formerly it was thought to be bacterial [3], to the use of genetic and antigenic information to inform vaccine development [4,5], diagnostics [6] and treatment selection [7]. Phylogeographic analyses combine sequence and geographic data to make inferences about the migration of influenza virus. Studies of influenza A/H3N2 show that China and South-east Asia are frequently the source of the lineages that then circulate globally [8-10].

What does this new level of detail offer to the infectious disease epidemiologist? Whereas the sequence of a single organism or clone can address questions about the microbe’s phenotype and history [11,12], comparisons of larger numbers of genome sequences can shed light on evolution and population genetics, using little other than the date of isolation in combination with sequence [13-16]. The combination of genome sequence data from clinical and environmental isolates and epidemiological data about the sources of the isolates can help characterize the origins, transmission, dynamics and evolution of infectious disease epidemics, with examples ranging from understanding how the pneumococcal population has evolved in response to use of antipneumococcal vaccination in children [14] to the sources and spread of the ongoing Ebola epidemic in West Africa [17]. In this review, we discuss the importance of these tools by first considering the conceptual advances in use of pathogen genome sequences, then addressing the applications of genomics-based methods for answering specific questions in infectious disease epidemiology and the associated research questions and methodological constraints that arise. Finally, we discuss policy and logistical and technological obstacles to achieving a potential transformation of public health microbiology.

## Conceptual advances in the use of pathogen genomics for infectious disease epidemiology

Historically, epidemiological monitoring of infectious diseases relied on case counts from clinical diagnosis, and sought to turn data about the infected populations into inferences about where and how the infectious disease spread. The most famous example is from the 19th century, in which John Snow mapped the locations of clinically defined cholera cases in an outbreak in London and inferred that the outbreak was due to contaminated water from the Broad Street pump; this was before identification of *Vibrio cholerae* as the etiologic agent. The epidemiologist’s line list (Table 1) aims to capture critical information about the demography, exposures and clinical features of the infected individuals that can then inform hypotheses about the nature and dynamics of disease transmission; for example, in the case of cholera in 19th century London, the geographic location of cases with respect to their water supply was used; however, more general characteristics, including age, gender and date of diagnosis, are among features that can be used to generate and test hypotheses about disease transmission or population susceptibility.

**Table 1:** **Example of a line list**

	**Demographic information**			**Clinical data**				**Diagnostic data**			**Exposure data**		**Microbial sequence** ^**a**^
**Case identifier**	**Age**	**Gender**	**Home location**	**Presenting symptoms**	**Date of onset**	**Underlying medical conditions**	**Clinical outcome**	**Specimen type**	**Diagnostic test**	**Result**	**Contacts** ^**b**^	**Exposure** ^**c**^	
1													
2													
3													
4													
5													
6													
7													
8													
9													
10													

Line lists are used in epidemiological investigations. The data fields here are examples of the types of information collected from each case. The fields are adjusted on the basis of the specific disease or syndrome under investigation. As sequencing of microbiological samples becomes part of routine clinical and public health microbiology practice, microbial sequence will become part of the line list data. ^a^Longitudinal time points, deep-sequencing, single colony, multiple colonies, and so on; ^b^for example, for communicable disease; ^c^for example, foods eaten for food-borne outbreak.

Advances in diagnostic tools have led to a more refined understanding of the dynamics of many infectious diseases by typing the pathogens by a genetic or phenotypic feature and adding these data to the line list (Table 2). Influenza again provides an illustrative example. Whereas during the 1918 influenza pandemic, the etiology of influenza was unknown (and mistakenly attributed to Pfeiffer’s bacillus, now called *Haemophilus influenzae*), we now have tools to confirm that an individual’s infection is caused by influenza virus, and further to characterize it by viral type, of which there are two relevant to human disease, A and B, and by subtype, defined by hemagglutinin (H) and neuraminidase (N), with examples including A/H3N2, A/H1N1 and A/H5N1. These data have clinical and epidemiological significance. Clinically, they aid in guiding treatment and prevention plans and in the development of novel diagnostics and therapeutics - for example, in 2009, recommended antiviral treatment regimens varied depending on whether an individual was infected with influenza A/H1N1, influenza A/H3N2 or influenza B [7]. In the area of prevention, development of effective vaccines depends now on the identification of antigenic variants within each subtype and construction of vaccines targeted to these antigenic variants [18]. Epidemiologically, rather than grouping all individuals with clinical influenza as the same, these tools have aided in understanding the evolutionary and epidemiological dynamics of influenza lineages [8-10,19,20], as well as the different profiles of mortality caused by each subtype [21]. Ironically, recent efforts to create a universal influenza vaccine effective against all subtypes may obviate some of the public health need to track individual subtypes [22]. Yet, if successful, the development of such vaccines will have depended on extensive studies of vaccine immunogenicity and protective efficacy against defined serotypes.

**Table 2:** **Time line of a number of key technological and scientific advances in infectious disease classification**

**Date**	**Advance**	**Applications**
1670s	Microscope invented by Leeuwenhoek	Visualize bacteria, protozoa
1850s	Puerperal fever identified as infectious and interventions implemented by Semmelweis [23]	Hospital infection control motivated by growing understanding of microbial etiology
1864	Cholera transmission by water proven by Snow	Risk factor (mode of transmission) and prevention measure for specific infectious syndrome
1890s	Proof of parasitic origin (Grassi) and mosquito transmission (Ross) of malaria	Vector control
1890s	Identification of microbial etiologies for tuberculosis, anthrax, and so on; Koch’s postulates	Targeted diagnostics, therapeutics, and move from syndromic diagnosis to pathogen identification
1900-1930s	Discovery of filterable animal viruses [24]	Influenza etiology settled (previously thought bacterial) [25]
1910s-1950s	Phenotypic subspecies taxonomy: serotyping [26,27], phage typing [28]	Association of particular types with prognosis [27,29], drug resistance
1944	Discovery of DNA as the genetic material [30]	Basis for genotyping tools for molecular epidemiology
1970	Restriction enzymes [31]	Basis for restriction fragment length polymorphism approaches, including pulsed field gel electrophoresis
1975-1985	Sanger DNA sequencing [32], PCR [33]	Basis for variable number tandem repeat (VNTR) and multilocus sequence typing (MLST) approaches to characterize microbes and their genetic relatedness
2000s-now	High-throughput rapid sequencing technologies	Microbial genome sequencing

Another phenotype that has been useful in monitoring and responding to clinically important pathogens is their pattern of susceptibility and resistance to a panel of antibiotics, with examples including methicillin-resistant *Staphylococcus aureus* (MRSA) and carbapenem-resistant *Enterobacteriaceae*, each of which has been associated with higher morbidity and mortality than drug-susceptible strains [34-36]**.** Other phenotypic approaches, such as serotyping, are shown in Box 1. Over the past several decades, genotypic approaches have supplemented phenotypic approaches to microbial identification and typing (Figure 1). In the 1990s, multilocus sequence typing (MLST) [37,38] and various restriction-pattern based approaches such as pulsed-field gel electrophoresis (PFGE) [39,40] and Southern-blot-based methods [41] defined pathogen isolates by small segments of their genomes. MLST, for example, helped to characterize the diversity of *Neisseria meningitidis*, to confirm that meningococcal disease is caused by a small number of invasive lineages, and to track these lineages as they spread geographically [38]. PFGE forms the basis of PulseNet [42], which uses this tool to detect food-borne pathogen outbreaks, linking cases caused by closely related bacteria that might not otherwise have been seen as part of an outbreak (publications using PulseNet have been collated [43]).

Each of the approaches described above aims to use characteristics of the microbial pathogens to better define the specific population responsible for a given outbreak, and thereby improve public health and clinical responses. However, these approaches employ a fraction of the data that could be used to resolve among isolates. In particular, they can classify isolates as indistinguishable, closely related or very different, with only rough estimates of the rate at which such genotypic markers diverge over time (Figure 1). Moreover, all of these methods gain their signal from a small fraction of the genome, so degree of similarity by these methods may not reflect overall similarity of the genomes, especially in pathogens that undergo frequent recombination, such that genome segments may have varying histories [44,45]. For this reason, direction and timing of evolutionary changes were difficult to infer using older techniques, and detailed phylogenetic inference was therefore impossible. As discussed below, many, though not all, of the advances possible with pathogen genomes build on the ability to infer phylogenies from genome sequences.

Genome sequencing and statistical tools based on molecular evolutionary theory have led to conceptual leaps over these prior typing schemes. Genome sequencing enables discrimination of pathogen isolates at the single nucleotide level, essentially providing a genome-level typing tool that serves the same purposes as earlier typing tools, but with much higher resolution. However, the biggest advances with pathogen genome sequences are their application to address three broad sets of questions that were difficult or impossible to answer with lower-resolution molecular epidemiological tools that were poorly suited to phylogenetic inference. First, analysis of sequences from samples collected longitudinally and from multiple sites over the course of an infection can address the nature of variation and evolution within a single infection, which occurs in bacterial, viral and parasitic infections yet was often undetectable by earlier typing methods [46]. Second, phylogenetic reconstructions from multiple pathogen genome sequences can be used to infer the rates and routes of transmission [47-49], providing information about the underlying contact networks that led to these transmissions [50]. Whereas older methods could categorize pairs of isolates as indistinguishable, closely related but distinguishable, or distantly related, single-nucleotide polymorphisms between whole genome sequences provide a nearly continuous scale of distance between isolates that offers the possibility of inferring the direction and routes of transmission, while identifying changes associated with this transmission history. Finally, sequence data can provide much more detailed information on medium to long-term microbial evolution, including variation in gene content and evidence of selection under pressures from interventions, such as vaccines, and changing niches [14,44]. Moreover, the development of so-called phylodynamic methods, largely based on coalescent theory from population genetics, has shown that a set of sequences from one point in time contains information about historical changes in the population size of the pathogen, which aids inferences about the dynamics of past transmission, that are independent of real-time case counting [51,52].

These advances can help address the following key questions that are of concern to the infectious disease epidemiologist (see Box 2):

1. Is there an outbreak?
2. Where, when and how did a pathogen enter the population of interest?
3. How quickly is the number of infections from the pathogen growing (that is, what are the epidemic dynamics)?
4. How is the pathogen spreading through the population?
5. What genes or genotypes are associated with the pathogen’s virulence or other phenotypes of interest?

In the sections below, we discuss the application of genome sequencing to these questions. We reference select examples, when available, of how pathogen genomics has been used to ask these questions. We note this review is not an exhaustive catalog of pathogen genomics efforts, as new and high-quality studies are being published routinely, but instead it aims to highlight illustrative examples. As the use of genomics, together with traditional epidemiological data sources, raises not just the conceptual advances described above, but also methodological challenges and constraints, we also highlight these challenges.

## Application of genome sequencing to key questions in the epidemiology of infectious diseases

### Identifying outbreaks

The term ‘outbreak’ generally refers to an elevation in disease incidence above background levels, and in more specific cases the term can refer to the emergence of a previously unrecognized pathogen such as Ebola in 1976 [53], HIV in the early 1980s [54,55], severe acute respiratory syndrome (SARS) in 2003 [56] or more recently Middle East respiratory syndrome coronavirus (MERS-CoV) [57]. The term can also refer to the initial entry of a pathogen into a community, such as cholera, which appeared in Haiti in 2010 [58,59]. Outbreaks are most frequently caused by the transmission of a clonal lineage of a pathogen, through a combination of limited initial diversity and population bottlenecks in transmission. Additionally, although rarely, outbreaks may also be caused by multiple lineages or pathogens; these mixed outbreaks may reflect co-circulating strains, such as influenza [60], a common source of contamination, such as the salmonella and campylobacter outbreak [61], ‘epidemic plasmids’ [62], or common modes of transmission [63]. Determining the presence of an outbreak, and whether or not it is clonal, can then help direct the response to abort it, as well as to prevent future outbreaks [64].

Several studies have used microbial genomics to determine whether a set of cases represents an outbreak by determining the phylogenetic relationship among outbreak cases to determine their relationship; isolates that are associated with a disease outbreak are often closely related based on background population structure. Examples of such studies include identifying the clonality of temporally and spatially linked hospital-based cases of infections with MRSA [65], carbapenemase-producing *Enterobacter* [66] and vancomycin-resistant enterococcus [66]. A study of tuberculosis demonstrated the potential utility in using genome sequencing to support both known and unknown links among infected individuals in transmission chains, and to help identify those likely not part of an outbreak [67]. In a genome-sequencing-based study of *N. meningitidis* from sporadic infections, epidemiologically unlinked cases were shown likely to be unrelated (reflecting population diversity, rather than the clonality expected from an outbreak) [66].

Interpretation of the phylogenetic relationships defined by whole genome sequencing depends on understanding the extent of diversity in the background population, the population dynamics and amount of diversity within an infected host, the population bottleneck in transmission events, and the epidemiological findings associated with each infection [64,66]. These background factors might differ depending on features of the infectious disease, including the mode of transmission (for example, contact-based, respiratory, food-borne or vector-borne), the extent of asymptomatic infection or carriage, and the duration of infection. As more studies investigate microbial population structures and dynamics, as well as examining the factors that influence them through experimental systems and large-scale genomic and metagenomic clinical and environmental surveys, the ability to assess the confidence of inferring epidemiological relationships based on genome data will improve.

### Determine the origin of an outbreak

The outbreak of a novel pathogen or the first entry of a known pathogen into a location prompts questions about its origin. The ability to pinpoint when and where an outbreak began depends on how representative existing case reporting is, as well as on knowledge of the population structure of the pathogen. In an ideal scenario where all known cases are reported, determining the origin of an outbreak is trivial. In reality, surveillance systems and case reporting are incomplete. In these circumstances, the use of sample collection time-stamps, where ‘time-stamp’ refers to the date when a sample was collected, in reconstruction of the phylogeny can aid in estimating the date of the most recent common ancestor (MRCA) of the pathogens sampled from infected individuals, which must by definition be no older than the origin of the outbreak. Additional demographic information about the isolates, such as geographic location, can contribute to estimating the characteristics of the MRCA and improve understanding of the modes of spread of the pathogen in question [68-73]; a recent study, for example, uses such data to infer the roots of the HIV epidemic [73].

Phylogenetic inference addressing questions about the origins of an outbreak requires background data that scale with the desired resolution of the answer. When the genome of *V. cholerae* from the outbreak in Haiti was placed into a phylogenetic context, it was reported that it was most closely related to a recently isolated strain from South Asia [58,59]. The more densely sampled the global population of the pathogen, both temporally and geographically, the greater the confidence in the inferences from the data. The availability of a larger number of *V. cholerae* genomes from the outbreak in Haiti, over several years [59], helped to improve the estimation of the MRCA and support the epidemiological hypothesis that there was a single introductory event that took place in early autumn of 2010.

The ongoing Ebola crisis illustrates both the challenges and promise of addressing questions about the origin of an outbreak. Whereas genome sequences of the Ebola virus from current and past outbreaks could be placed into a phylogeny to guide inference about its appearance for the first time in West Africa, the samples and the details of constructing the phylogeny can influence the conclusions, such that differing phylogenies emerge from inclusion and exclusion of intergenic regions [74,75]. Large-scale sequencing of patient samples can help confirm epidemiological conclusions that this outbreak had a single origin [17]. The fact that only patient but not environmental samples are available deepens the mystery of the natural ecology of Ebola virus, and raises questions about the population structure of the environmental reservoir, and about the extent to which human outbreaks are the products of rare exposure or rare adaptation of Ebola virus to human hosts.

There are important caveats to the use of phylogenetic models for inferring the origin(s) of a disease outbreak. For example, the sensitivity of phylogeographic and phylodemographic analyses remains unclear. As methods develop to link phylogenetic reconstructions with geographic and demographic information, it is important to be aware of the uncertainty in phylogenetic models. Recent reviews discuss such methods and their utility in epidemiological inference [52,76,77] and challenges in their use [78].

A further caveat to the use of these data comes from sampling biases and the risk of interpreting the resulting phylogenies as if they are representative of an entire pathogen population. Interpretation of phylogenies benefits from characterizing the extent of asymptomatic infection, which can influence the inference about the epidemiological scenarios that gave rise to the outbreak; the more unseen and unsampled transmitters, the more difficult to accurately reconstruct transmission [79,80]. Gaps in geographic and temporal sampling will contribute to uncertainty, suggesting that pathogens with extensive asymptomatic and environmental or vector reservoirs may face particular challenges that constrain the resolution and confidence of phylogeny-derived estimates. The greater the extent of uncharacterized disease and, correspondingly, the greater duration of infection, rate of diversification and transmitted diversity, the more uncertainty in phylogeny-based inferences [81].

### Calculate epidemic parameters

The epidemic growth rate and reproduction number (*R*) are related measures of how contagious a pathogen is; these measures guide risk assessment and interventions for many infectious diseases, particularly emerging diseases [82]. Formally, the reproduction number is the number of cases on average caused by a single infected individual over the course of the individual’s infectious period, and the epidemic growth rate refers to the proportional increase in the number of cases per unit time. Gene genealogies have been used in estimating HIV’s generation time [83], and the basic reproductive number of hepatitis C virus (HCV) [84]. For infections whose incidence and prevalence are difficult to observe directly due to high fraction of asymptomatic, subclinical or unreported infection, inferences based on pure sequence data may be usable to infer the effects of mass vaccination in reducing transmission [85].

In the early phase of an outbreak, when case detection may be highly imperfect and nonrandom, molecular clock estimates of time to the most recent common ancestor can estimate the growth rate of the pathogen population in a way that is partially independent of methods that rely on ongoing case-ascertainment. Within months of the emergence of the influenza strain pH1N1 in 2009, analysis of the phylogeny using an evolutionary model with exponential growth provided an estimate of the growth rate, and, together with the assumption that pH1N1 had the same generation time as other influenza infections, the reproductive number [86]. Phylogenetic analysis can also provide qualitative insights into epidemic parameters: early analysis of MERS-CoV has offered an initial glimpse of the pandemic potential of this pathogen, with interpretation of clade disappearances as possibly reflecting an *R*_0_ less than 1 [80] (where *R*_0_ is the ‘basic reproductive number’, referring to the average number of infected individuals caused by a single infectious person in an entirely susceptible population). A feature of these approaches is that they do not require (and in some cases cannot even use) dense sampling of most cases from an outbreak, only representative sampling of a fraction of cases at one or more time points.

Integration of epidemiological models and phylogenetic reconstructions to infer epidemic parameters, including *R*_0_, transmission rates and population size, is an exciting and active area of research [52,87-89]. Although work to date has focused on using these tools with rapidly mutating RNA viruses, including HIV, HCV and dengue, development of statistical approaches that consider the relationship between parameters such as the serial interval (the average time between infection and subsequent transmission), duration of infection, and sampling of the lineages in an individual and the within-host diversity, among others, will be needed to explore generalizing these approaches.

### Track and reconstruct transmission routes

Understanding transmission routes is essential in the control of infectious diseases. Studies that reveal who infected whom can help to identify a pathogen’s mode of transmission and thereby direct infection control and prevention policies to prevent further disease spread [65,90,91]. At broad temporal or spatial resolution, tracking transmission can identify clusters of related cases and reveal patterns of pathogen spread; this allows inferences about the structure of the underlying network along which a pathogen spreads [92]. Accumulated experience from the study of multiple outbreaks can then help understand the common patterns for particular pathogens or populations; as the transmission patterns for more outbreaks are described, commonalities - for example, the extent to which superspreaders are important - may help lead to more effective public health interventions.

A range of approaches recently developed to reconstruct transmission at a detailed level involve statistical analyses that formally combine evidence of genomic relatedness between pathogens isolated from different hosts, with temporal, geographic and other data to arrive at inference of likely transmission trees. In one innovative example, spatial and temporal data were combined with genomic data to estimate the spread of H7N7 influenza among farms in the Netherlands, and then a meteorologic data set was overlaid to test the hypothesis that wind direction explained patterns of spread [49]. Results were consistent with this hypothesis, illustrating two general points: first that genomic data can contribute to identifying a new transmission mechanism, which in this case was wind-borne transmission of influenza, and second that as our understanding of transmission mechanisms grows, the appropriate metadata to combine with our analyses will also grow and be pathogen-specific in some cases. Some of these approaches, particularly those that wish to reconstruct individual transmission events, require dense sampling of most of the cases in an outbreak, and can be complicated by factors that limit or bias sampling, including undetected unknown or difficult to access reservoirs, including asymptomatic and vector-borne infections. Other approaches, which focus on less granular inference, such as transmission from one sexual mixing group or city to another, without interest in the individual involved, can be applied to much sparser samples. Importantly, recent work has also emphasized the limits of inference of transmission from genomic data alone and indicated that it can both help motivate and substantiate traditional epidemiological efforts and conclusions [48,93].

### Identify genes and genotypes associated with pathogen phenotypes of interest

Traditionally, surveillance has been a largely separate activity from functional genetic analysis of pathogens. As sequence data become more fully integrated into surveillance, it becomes natural to ask how far the functional and phenotypic interpretation of such data can be pushed, from identifying putative virulence factors by the presence or absence of a gene [94] to performing genome-wide association studies (GWAS) using large numbers of isolates [95]. For the epidemiologist, this also provides genetic signatures of specific phenotypes - such as resistance or virulence - that can be tracked in the context of routine surveillance, monitoring of strains and development of new diagnostics.

Initially, phenotypic data, including virulence and drug-resistance phenotypes, have to be collected alongside sequence data to assemble the database from which correlations between genotype and phenotype can be observed. Classical genetic studies can then test hypotheses about which of these observed correlations are causal. Those that are suggest the opportunity to develop new diagnostic and prognostic tests based on sequence data alone and to suggest further hypotheses about pathogen biology and host-pathogen interactions that can direct additional experiments.

This approach has three requirements. First, it requires standardized and reproducible genomic assemblies and annotations or access to the raw reads for each of the isolates so that uniform tools can be applied to analyze genotype-phenotype relationships. Second, it requires reporting of the key phenotypic data, including clinical data, for microbial GWAS to search for pathogen determinants of clinical manifestations. For optimal scientific and public health outcomes, such data should be stored in standardized fashion and should be available for study, regardless of whether the original analyses are done by individual institutions with ‘in-house’ sequencing and bioinformatics expertise or through ‘send-out’ testing to companies that report genotype and phenotype information. Third, the use of genotype to replace culture and phenotypic testing requires caution, given that linkage, epistasis and other processes may weaken the strength of the genotype-phenotype association over time. The emergence and spread of a *Chlamydia trachomatis* variant in Sweden characterized by a deletion in the locus targeted by a commonly used nucleic acid amplification diagnostic test offers one related cautionary tale [96]. Even in the context of an experimentally established causal genotype-phenotype relationship, repeated validation over time will be required as, for example, alternative genetic bases for the phenotype may appear in the population.

Whereas many properties of an infection may be predictable from pathogen genotype alone, assessment of change in pathogen populations in response to large-scale interventions, such as pneumococcal vaccination, provides an opportunity to monitor the ecological response of microbial communities and the interplay between hosts and pathogens [14]. Studies of niche differentiation suggest a key new direction for understanding and modeling infectious disease transmission, building on prior work that uses serotypes to consider the heterogeneity in which pathogens infect which people. To date, heterogeneity is mostly considered in terms of acquired immunity or proxies for it, such as age. Studies such as the age-stratification of pneumococcal gene content [14] suggest signatures of interplay between host immunity and pathogen evolution. Vaccine escape is one of the most important manifestations of these interactions; deepening characterization of the immune responses of hosts in which escape mutants arise and transmit most successfully offers a particularly exciting and developing field [97]. This is particularly high risk/reward as many hypotheses may be wrong, but so far we have modeled spread of particular species largely without regard to heterogeneity of which pathogen infects which person.

## Implementation of microbial genomics in public health: challenges and opportunities

Individual studies that demonstrate the potential for pathogen genome sequences to contribute to infectious disease epidemiology and public health make a compelling case for incorporating these data into standard practice; however, the implementation presents a number of challenges and opportunities.

### Database and analytical development

As databases grow in sequence and metadata, and ideally incorporate the dates and locations of sample collections, as well as the method of isolation of the sequenced samples from the environment or infected individual, rapid integration of new data may permit automated identification of outbreaks and inferences about their origins. A system that recognizes the appearance of samples more closely related than expected based on what is known about the population structure and incidence could accelerate outbreak identification and facilitate responses. Further, by maintaining a database of samples that describe the ecology of a pathogen and the background population diversity, it may also be easier to place a clinical specimen into a phylogeny to infer its origin and identify the existence of an outbreak. For example, the time taken to discover an outbreak spread across locations, such as a food-borne outbreak in which the contaminated items are shipped to a broad geographic area, could be improved [98]. Incorporation of sequence data in routine disease surveillance could help shed light on the transmission dynamics of pathogens, and thereby guide public health interventions. The Global Microbial Identifier project [99] and similar efforts aim to address the challenges of generating a uniform database of microbial sequences and associated metadata, though the technical and political obstacles to universal uptake are formidable.

The role of microbial genomics in public health and clinical microbiology raises critical questions about infrastructure development and training personnel who bridge understanding of the subtleties of the infectious diseases they study with familiarity with genomics and bioinformatics techniques. Laboratories interested in developing their own sequencing platform will have to invest in one of the available technologies, and, as of now, develop in-house solutions to data processing, analytics and interfacing with public databases. This will require some combination of hiring bioinformaticians and providing training to clinical microbiology and public health laboratory staff. Similarly, infectious disease epidemiologists who will be asked to incorporate genomic data into their routine practice will need background in genomics and associated methods and theory as well as skills in processing and managing these data sets. Further, as the field is rapidly evolving technologically and computationally, the creation of ‘gold standard’ approaches for clinical and public health practice will likely need frequent updating.

### Data sources

What sets of data should be included in these databases? Infectious disease epidemiological studies draw on routine surveillance projects, outbreak investigations, and research studies. The addition of pathogen genome sequences is a natural extension to these studies that helps achieve their goals. Another potential source of data comes from the clinical microbiology laboratories that, for the most part, do not publish or make available data on the types and numbers of microbes identified from patients. With clinical microbiology laboratories taking up microbial genome sequencing [100], there are remarkable and potentially transformative opportunities for vastly expanding the data streams available for understanding infectious disease dynamics and microbial ecology and evolution, including the emergence and spread of antimicrobial resistance. As the technology and tools for bringing pathogen genome sequencing into clinical realms develops, it is worth following the models of efforts to monitor antibiotic resistance (for example, WHONET [101], EARS-Net [102]) for specific or, ideally, for all clinically isolated pathogens and exploring ways to include and automate uploading these data to public health microbiology databases.

The potential contributions from such a vast expansion of available public health and microbiological data make it important to consider the associated questions. If sequencing of clinical samples becomes a routine part of clinical care or local infection control, should there be an obligation for clinical laboratories to upload their data (stored in a wide range of electronic medical records systems) to a uniform public health database? What data, and for what pathogens? If sequencing is not part of routine clinical care or local infection control, then what pathogens should be sequenced, by whom and with what funding? Will the growing consortium of public health agencies, academics and industry recommend standardized sequencing and analytic methods to facilitate integration of data from across multiple institutions? If so, whose job should it be to generate and maintain the standards in this rapidly developing field? There will be false positives for any algorithm that is intended to detect outbreaks; what false-positive rate will be acceptable? Who will have the responsibility for following up possible outbreaks? Failure to include clinical microbiological samples and data, and failure to develop standards that allow for temporal and geographic aggregation of data, will represent a huge missed opportunity for advancing infectious disease epidemiology and public health.

### Privacy and legal concerns

A critical question in the integration of genomics into public health microbiology is to understand what extent data should be available to researchers and the public. This has institutional and infrastructure implications for how the metadata that accompany the microbial genome sequences should be collected and stored. Ideally, metadata, including microbiological phenotype profiles of antibiotic resistance, and patient-centered data on host demographics and clinical course, would be readily accessible for automated analyses or for directed research investigations. However, it is worth noting that collection, storage and use of patient-centered data raises privacy and security issues that will need to be addressed. This also raises medical-legal scenarios, depending on availability of data and on confidence in the conclusions: when is action to investigate a potential outbreak warranted, and when is it obligatory?

### Funding

As described above, there are many emerging research questions related to transforming public health microbiology through the use of genome sequencing and analysis. Traditionally, genome sequencing and other sophisticated laboratory-based technologies have been the province of funding bodies and research groups devoted to basic biomedical science, while the detection and characterization of outbreaks, along with routine surveillance, have been the province of epidemiologists and others specializing in applied public health. In the application of a now established technology to answer questions at the population level, cooperation between these groups is essential, both to ensure that a promising transdisciplinary approach does not fall through the cracks between funders with priorities on one side or the other of the basic biology-epidemiology divide, and to ensure that the best technology is married with the best quantitative and analytical tools at stages from study design and data collection through analysis and inference.

## Conclusions

To date, studies as described above have demonstrated the potential for an expanded line list of data that include genome sequences to augment epidemiological inquiry and generate inferences about the spread and evolution of pathogens, to help guide efforts to reduce disease burden. Recent incorporation of pathogen genome sequencing into the efforts of Public Health England [103] and emphasis on the importance of a public health surveillance and response system based on pathogen genomics in the recent report from the President’s Council of Advisors on Science and Technology in September 2014 on combating antibiotic resistance [104] foreshadow the large-scale adoption of pathogen genomics into the public health infrastructure. Maximizing impact will require basic and applied research efforts to develop the methods, databases, analytics and platforms to go from samples to actionable public health data, and the creation of a flexible system that can test and incorporate novel epidemiological approaches.

For most pathogens, there are fundamental aspects of microbial diversity in human hosts and the environment that we do not yet understand but which bear directly on epidemiological questions. Foundational work is needed at many levels, including: description of genetic diversity over the course of an infection and in transmission, first under ‘typical’ conditions and, over time, with a more sophisticated understanding of the impact of other factors on this diversity, such as microbiome, immunocompromised status, duration of infection, route of transmission, level of symptomatic disease and other host characteristics [105]; defining the population structure of pathogens at multiple geographic, demographic and temporal scales; methodological advances in phylogenetic approaches that can integrate within-host and population diversity into statistical measures of confidence in reconstructions of transmission chains, and approaches to dealing with the impact of missing data on phylogenetic reconstructions and epidemiological inference. Advances in these fields, and in fields that study heterogeneity in host susceptibility, suggest exciting directions for improving public health efforts for infectious disease treatment and prevention.

## Box 1. Techniques for classifying microbes for epidemiological investigations

Phenotypic techniques

- Biotyping (for example, biochemical reactions, colony morphology)
- Serotyping
- Other typing tools (for example, bacteriophage, bacteriocin)
- Antimicrobial susceptibility

Molecular/genomic techniques

- Restriction fragment length polymorphism (for example, pulsed-field gel electrophoresis)
- Multilocus sequence typing
- Genome sequencing

## Box 2. Using pathogen genomics in infectious disease epidemiology

Pathogen genome sequencing can impact the study of infectious diseases epidemiology through contributions to the following questions:

- Is there an outbreak?
- When/where was the origin of the outbreak?
- What is the growth rate and reproduction number?
- What is the transmission chain (at the level of individuals or populations)?
- What genes and genotypes are associated with both pathogen and clinical phenotypes of interest?

Addressing each of these questions, however, is not as simple as just comparing the sequences of clinical isolates. Key areas of both theoretical and experimental investigation that may be needed to answer the questions and describe the confidence in those answers include:

- The microbial ecological diversity/population structure at the appropriate scale for the outbreak question
- The genomic diversity in a single infection, how dynamic this diversity is over the course of an infection/colonization, and how much of this diversity is transmitted
- The extent of gaps in geographic and temporal sampling and the potential of asymptomatic infection to contribute to uncertainty
- Uncertainty in phylogenetic models such as that deriving from sampling biases and factors influencing determination of molecular clock rate

Bringing these methods to public health microbiology infrastructure poses its own set of challenges and opportunities. These range from developing the databases and methods for storing and analyzing line-list data that include pathogen genome sequences, determining the logistics of data sources and sharing and interpretation and follow-up of results, and determining which agencies will fund the fundamental research that will help this field grow as well as transition into a flexible and modern system of public health microbiology.

## Abbreviations

GWAS: genome-wide association study
HCV: hepatitis C virus
MERS-CoV: Middle East respiratory syndrome coronavirus
MLST: multilocus sequence typing
MRCA: most recent common ancestor
MRSA: methicillin-resistant *Staphylococcus aureus*
PFGE: pulsed-field gel electrophoresis
